# Fornix Integrity Is Differently Associated With Cognition in Healthy Aging and Non-amnestic Mild Cognitive Impairment: A Pilot Diffusion Tensor Imaging Study in Thai Older Adults

**DOI:** 10.3389/fnagi.2020.594002

**Published:** 2020-12-02

**Authors:** Patcharaporn Srisaikaew, Nahathai Wongpakaran, Nicole D. Anderson, J. Jean Chen, Suchart Kothan, Pairada Varnado, Kittisak Unsrisong, Pasuk Mahakkanukrauh

**Affiliations:** ^1^Ph.D. Program in Anatomy, Faculty of Medicine, Chiang Mai University, Chiang Mai, Thailand; ^2^Department of Anatomy, Faculty of Medicine, Chiang Mai University, Chiang Mai, Thailand; ^3^Geriatric Psychiatry Unit, Department of Psychiatry, Faculty of Medicine, Chiang Mai University, Chiang Mai, Thailand; ^4^Rotman Research Institute, Baycrest Health Science, Toronto, ON, Canada; ^5^Department of Psychology and Psychiatry, University of Toronto, Toronto, ON, Canada; ^6^Department of Medical Biophysics, University of Toronto, Toronto, ON, Canada; ^7^Department of Radiologic Technology, Faculty of Associated Medical Sciences, Chiang Mai University, Chiang Mai, Thailand; ^8^Department of Radiology, Faculty of Medicine, Chiang Mai University, Chiang Mai, Thailand; ^9^Excellence in Osteology Research and Training Center (ORTC), Chiang Mai University, Chiang Mai, Thailand

**Keywords:** non-amnestic mild cognitive impairment (n-aMCI), fornix, diffusion tensor imaging (DTI), fractional anisotropy (FA), fiber tract length (FTL), cognitive performance, executive function, vascular dementia (VaD)

## Abstract

Damage to the fornix leads to significant memory impairment and executive dysfunction and is associated with dementia risk. We sought to identify if fornix integrity and fiber length are disrupted in mild cognitive impairment (MCI) and how they associate with cognition. Data from 14 healthy older adult controls (HCs) and 17 subjects with non-amnestic MCI (n-aMCI) were analyzed. Diffusion tensor imaging (DTI) at 1.5 Tesla MRI was performed to enable manual tracing of the fornix and calculation of DTI parameters. Higher fractional anisotropy of body and column of the fornix was associated with better executive functioning and memory, more strongly in the HC than in the n-aMCI group. Fornix fiber tract length (FTL) was associated with better executive function, more strongly in the n-aMCI than in the HC group, and with better memory, more strongly in the HC than in the n-aMCI group. These results highlight a decline in the contributions of the fornix to cognition in n-aMCI and suggest that maintenance of fornix FTL is essential for sustaining executive functioning in people with n-aMCI.

## Introduction

Approximately 60% of the world's population lives in the Asia-Pacific region, where the prevalence of dementia is expected to rise from 23 million in 2015 to 71 million in 2050 (Venketasubramanian et al., 2010; Alzheimer's Disease International, 2014, 2018, 2019), among which vascular dementia (VaD) is more prevalent than in western populations. Mild cognitive impairment (MCI) is generally considered the transitional state between healthy aging and dementia (Petersen et al., 2001; American Psychiatric Association, 2013; Anderson, 2019). The criteria for MCI (termed minor cognitive disorder by the American Psychiatric Association) include concerns about changes in cognition, impairment in one or more cognitive domains, preservation of independence in functional abilities, and no dementia (American Psychiatric Association, 2013). People with MCI can be categorized as amnestic (aMCI) or non-amnestic (n-aMCI). aMCI is likely to progress to Alzheimer's disease (AD), whereas n-aMCI most typically develops into other types of dementia, prominently into VaD but also into frontotemporal dementia (FTD) or Lewy body dementia (LBD), but can also progress to AD (Petersen et al., 1995; Farlow et al., 2004; Petersen, 2004). VaD is the second most common cause of dementia after AD, causing 20–30% of global dementia cases (Alzheimer's Disease International, 2014), 15–20% in North America and Europe (Plassman et al., 2007; Rizzi et al., 2014), and ~30% in Asia (Jhoo et al., 2008; Chan et al., 2013).

VaD is usually caused by decreased blood flow to the brain, with the risk of incident dementia within 5 years being 6.5 times higher after a stroke and 1.5 times higher after a transient ischemic attack (TIA) (Pendlebury et al., 2019). In VaD, white matter (WM) inflammation is associated with oxidative stress, cerebral hypoperfusion, and thromboembolism (Venkat et al., 2015). Clinical signs and symptoms of VaD depend on the cause of VaD, affected areas, and size of infarction. A decrease in cerebral blood flow (CBF) and hypoxia in the prefrontal cortex (PFC), basal ganglia, and hippocampus is typically associated with cognitive decline and behavioral changes in VaD (Iadecola, 2013; Venkat et al., 2015). In a recent study, patients with small- and large-vessel VaD showed dysfunction in memory, executive function, and attention domains (Sengupta et al., 2019).

A WM tract that plays a major role in supporting these functional domains is the fornix, a discrete bidirectional tract bundle that connects the hippocampus to other limbic structures that is crucial for normal cognitive function and is a subcortical component of the limbic system (Teipel et al., 2008; Christiansen et al., 2016; Rabin et al., 2019). As a part of the fornix extends from the hippocampal–diencephalic system, the fornix plays an important role in the Papez circuit (Papez, 1937). It is the major efferent pathway in the human memory circuit and is thought to be especially key for maintaining episodic memory (EM) (Thomas et al., 2011; Douet and Chang, 2015) and executive function (EF) (Sasson et al., 2013).

Damage to the fornix has been shown to lead to significant memory and cognitive impairment (Oishi et al., 2009; Thomas et al., 2011; Mielke et al., 2012; Fletcher et al., 2013; Wang et al., 2018; Metzler-Baddeley et al., 2019). Likewise, infarction of the fornix can lead to neurodegeneration of the fornix, cognitive function decline, and VaD or subcortical VaD (SVD) (Cummings, 1994; Kalaria and Erkinjuntti, 2006; Zhuang et al., 2013; Mugikura and Takahashi, 2015; Takano et al., 2018; Zhu et al., 2018). Neuropsychological evaluation demonstrated the existence of an amnesia syndrome with deficit of executive functions in patients with bilateral infarction of the fornix, especially in the anterior column of the fornix (Nestor et al., 2007; Rizek et al., 2013; Salvalaggio et al., 2018). Given these findings, we expected reduced fornix integrity in n-aMCI compared to healthy older adults and for fornix integrity to be related to memory and executive functioning performance.

Diffusion tensor imaging (DTI) has been used fruitfully to study *in vivo* WM microstructure in the human brain *via* voxelwise analysis, region-of-interest (ROI) analysis, or fiber tractography (FT) (Liu et al., 2009). The majority of DTI studies have revealed a reduction of fractional anisotropy (FA) and an increase in mean diffusivity (MD), also known as the apparent diffusion coefficient (ADC), with advancing age (Beaulieu, 2002; Peters, 2002; DeBoy et al., 2007; Lebel et al., 2008; Mamere et al., 2009; Klawiter et al., 2011; Aung et al., 2013). These findings have been attributed to the breakdown of the myelin sheath and axonal membrane degradation such as axonal disintegration, oligodendrocytosis, astrocytosis, and Wallerian degeneration (WD) (Werring et al., 2000; Pierpaoli et al., 2001; Kiuchi et al., 2009; Kantarci et al., 2011; Dimitra et al., 2013). Indeed, abnormal fornix tissue cytoarchitecture has been associated with neuropathological abnormalities in those who are cognitively normal and later progress to MCI (Chao et al., 2013). Patients with MCI and/or AD show significant reductions of FA of the fornix, which highlights the importance of this key structure as an imaging marker to predict early disease progression (Liu et al., 2011; Thomas et al., 2011; Mielke et al., 2012; Pelletier et al., 2013; Yu et al., 2014; Metzler-Baddeley et al., 2019; da Rocha et al., 2020). Moreover, pathology of the fornix affects several brain networks with which it is interconnected (Nowrangi and Rosenberg, 2015).

In this study, we used DTI to assess the WM microstructure of the fornix in a cohort of Thai older adults. While the current literature has focused more on DTI markers of aMCI and AD and on whole-brain analyses, our aim was to specifically target the fornix, as its unique connectivity can shed light on the pathophysiology of n-aMCI, which is particularly prevalent in the Asia-Pacific region. Importantly, we approached this aim through both volumetric analysis and tractography. Our hypothesis was that WM integrity and fiber tract length (FTL) of the fornix would be sensitive to n-aMCI and would associate with cognitive functioning.

## Materials and Methods

### Participants and Study Design

Participants aged 60 years and older with no history of dementia, no active depression disorders, and normal levels of daily function were recruited through the Maharaj Nakorn Chiang Mai Hospital and the local community. This study received institutional ethical approval. All participants in this study had voluntarily offered to undergo blood collection, cognitive screening tests, neuropsychological battery testing by a geriatric psychologist, and MRI scan by a well-trained radiologic technologist. Participants provided written informed consent before beginning the study. Participants were excluded from enrollment if they had (1) a history of infection, infarction, or other focal lesions in a brain structure critically associated with memory; (2) alcohol or substance abuse or dependence within the past 2 years; (3) significant neurologic diseases within the past 1 year; active claustrophobia, hypothyroidism, hyperthyroidism, vitamin B12 deficiency, neurosyphilis [rapid plasma reagin (RPR) or Treponema pallidum hemagglutination (THPA) positive], or the human immunodeficiency virus (HIV); (4) current use of psychoactive medications; significant head trauma with post-traumatic loss of consciousness for at least 30 min at any point in their life; (5) loss of senses (blindness, deafness) or photosensitive epilepsy; presence of any metallic implants; and (6) any significant systemic illness or unstable medical condition that could lead to difficulty complying with the protocol.

Eighty participants were recruited, of which 39 were excluded after screening due to (1) 10 cases of mild anemia, (2) three cases of incomplete screening, (3) three cases of claustrophobia, (4) two cases of depression, (5) two cases of abnormal thyroid function, (6) two cases of obstructive sleep apnea, (7) two cases where participants were taking medications that affect cognition (i.e., prostatitis treatment), (8) one case of contracted syphilis, (9) one case of color blindness, (10) one case of generalized anxiety disorder, and (11) one case of low white blood cell count. Eleven other participants were excluded due to an unclear diagnosis after neuropsychological testing. Forty-one participants met the initial inclusion criteria, consisting of 20 healthy controls (HCs) and 21 with MCI. All of the participants with MCI met the criteria for non-amnestic MCI (n-aMCI), all presenting with executive dysfunction. Ten participants were excluded from the imaging analysis due to incomplete MRI acquisitions and/or atypical projection of the fornix. Therefore, data from 31 participants are presented. The resulting cohort consists of 14 HCs and 17 n-aMCI.

### Clinical Evaluation

Each participant received multidisciplinary clinical evaluations at the Geriatric Psychiatry Clinic, Maharaj Nakorn Chiang Mai Hospital. Evaluations included (1) a detailed medical history; (2) physical and neurological examinations; (3) medical blood tests including fasting blood sugar (FBS), lipid profile (cholesterol and triglyceride), complete blood count (CBC), blood–urea–nitrogen (BUN) and creatinine (Cr), blood electrolytes (sodium, potassium, chloride, bicarbonate, calcium, magnesium, and phosphorus), triiodothyronine (T3), thyroxine (T4), thyroid-stimulating hormone (TSH) levels, and vitamin B12 (cobalamin), the RPR test, TPHA test, and HIV testing by a well-trained HIV counselor; and (4) cognitive screening tests including Montreal Cognitive Assessment (MoCA) (Nasreddine et al., 2005; Hemrungrojn, 2011), Mini-Cog Test (Borson et al., 2000; Trongsakul et al., 2015), Thai Geriatric Depression Scale-15 (TGDS-15) (Sheik, 1986; Wongpakaran and Wongpakaran, 2012), and The Barthel Index for Activities of Daily Living (ADL). In order to proceed in the study, participants needed to pass the Mini-Cog Test (score ≥3) and TGDS-15 (score <6) and to have no abnormal blood work results indicating conditions that could affect cognition.

### Neuropsychological Testing

Subtests of the Wechsler Memory Scale-Third Edition (WMS-III) and the Wechsler Adult Intelligence Scale-Fourth Edition (WAIS-IV) were used to measure three cognitive domains: attention, executive function, and memory.

To correct for multiple comparisons, composite scores were calculated. A z-score was calculated for each participant's performance on each cognitive test relative to the mean and standard deviation across all participants. The z-scores were multiplied by −1 in cases where higher scores indicated worse performance and then averaged within the domain:

- Attention: Digit Span Test, Digit Symbol-Coding Test, and Trail Making Test (TMT) Part.
- Executive function: TMT Part B, Block Design test, Verbal Fluency test (Phonemic and Animal), and the Stroop Color and Word Test (SCWT).
- Memory: Letter-Number Sequencing Test and Word List Memory I and II.

Diagnosis of cognitively normal or MCI (minor neurocognitive disorder) was made according to the American Psychiatric Association's (APA) Diagnostic and Statistical Manual of Mental Disorders-Fifth Edition (DSM-5®) criteria (American Psychiatric Association, 2013) by a consensus conference of a geriatric psychiatrist and neuropsychologist.

### MRI Acquisition

All participants were scanned on a 1.5 Tesla MR Philips Ingenia system equipped with a 15-channel head/spine array coil at the Associated Medical Science (AMS) Clinical Service Center, Department of Radiologic Technology, Faculty of Associated Medical Sciences, Chiang Mai University. The examination protocol included: axial DTI, T2 weighted (T2W) imaging, fluid-attenuated inversion recovery (FLAIR), and T1 weighted (T1W) imaging. The DTI protocol used the following parameters: repetition time (TR) = 5.0 s, echo time (TE) = 90 ms, FOV = 224 mm, matrix = 128 × 128, 49 directions, slice/gap 5.0/1 mm, b-value = 0 (1 volume per acquisition) and 1,000 s/mm^2^ applied in 12 diffusion gradient orientations, and 75 slices. The total scan time was 25 min.

### Preprocessing

The DTI data were analyzed using the FMRIB (University of Oxford's Center for Functional Magnetic Resonance Imaging of the Brain) Software Library (FSL) Diffusion Toolbox FSL release 5.0.10 (https://fsl.fmrib.ox.ac.uk/fsl/fslwiki). All DICOM files were converted into NIFTI files using the MRICron utility dcm2nii (http://www.nitrc.org/projects/mricron), and the first volume (the b = 0 image) was used to generate a binary brain mask with a threshold of 0.2 by using Brain Extraction Tool (BET). Then, the DTI parameters FA, axial diffusivity (AxD), mean diffusivity (MD), and radial diffusivity (RD) were derived from each participant's preprocessed DTI data.

Non-linear registration to the FMRIB58_FA space was applied to align the individual FA maps into a Montreal Neurological Institute (MNI) 152 standard space (http://imaging.mrc-cbu.cam.ac.uk/imaging/MniTalairach). The mean FA image was created from across all participants to generate a mean FA skeleton, which represents the center of WM tracts shared by all participants. To exclude voxels containing peripheral tracts, partial volume effects with gray matter (GM), and cerebrospinal fluid (CSF), the mean FA skeleton voxel was thresholded at FA ≥ 0.2.

**Figure 1:**
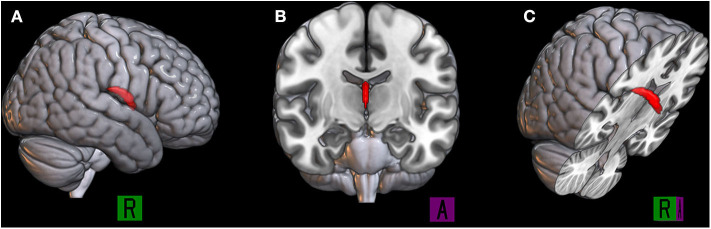
The binary mask of the body and column of the fornix is represented in red, the 3D rendered by using MRIcroGL (https://www.mccauslandcenter.sc.edu/mricrogl/) in the sagittal plane **(A)**, coronal plane **(B)**, and right oblique plane **(C)**.

Given the small size and high intersubject variability of fornix anatomy, in our group analyses, we used standard-space binary masks to isolate specific anatomical substrates of the fornix based on the JHU ICBM-DTI-81 WM Labels Atlas (Mori et al., 2005, 2008) (Figure 1). Subsequently, FSL's fslmeants (https://fsl.fmrib.ox.ac.uk/fsl/fslwiki/Fslutils) was used to extract the average time course of FA and ADC values over 2 JHU-atlas masks for each participant: the whole tract of the fornix (denoted by subscript “whole”) and the body and column (BC) of the fornix.

### Fiber Tract Length Measurement

**Figure 2:**
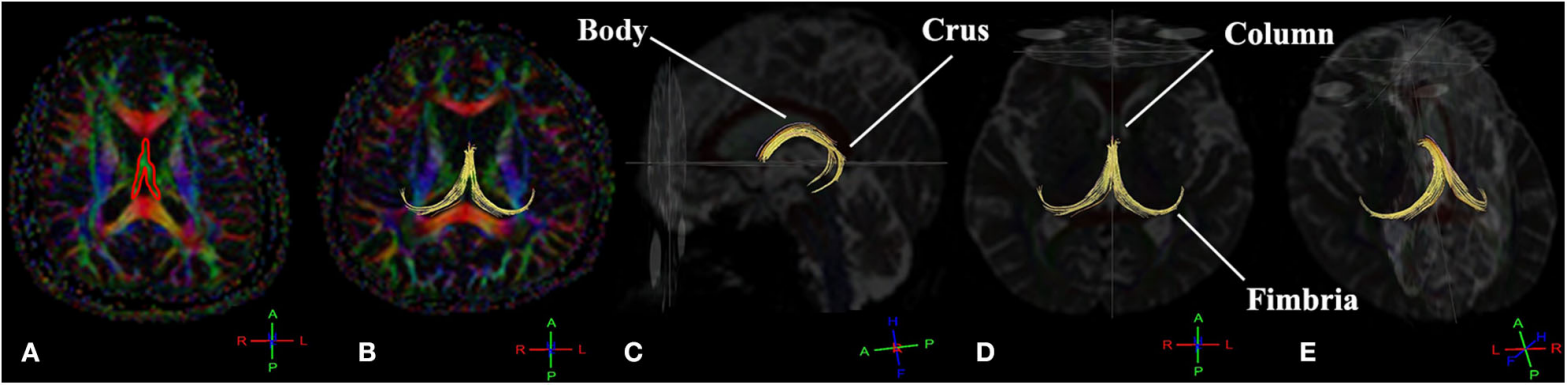
The deterministic tractography of the complete projection of the fornix tract (yellow tract) consists of fimbriae, crura, body, and column. The manual tracing region of interest (ROI) draws on the 2D first eigenvector fractional anisotropy (FEFA) map on the axial plane of the fornix **(A)**. The fornix fiber tract in the axial plane **(B)**, sagittal plane **(C)**, axial plane **(D)**, and left oblique view **(E)**. The conventional red-green-blue color-coding was used for display purposes (red for right–left, blue for dorsal–ventral, and green for anterior–posterior) (Müller and Kassubek, 2007).

We performed fornix tractography using the Phillips proprietary software, FiberTrak, which is based on the Fiber Assignment with Continuous Tracking (FACT) algorithm (Mukherjee et al., 2008; Christidi et al., 2016). This deterministic DTI fiber tracking technique was performed with an FA threshold of 0.2. The fornix was manually drawn on the axial plane based on anatomical knowledge on the color-coded first eigenvector FA (FEFA) map by an experienced radiologic technologist and by three raters: (1) a professional rater with experience with DTI of the brain and manual tracing, (2) an intermediate rater who knew about DTI but had no experience with the protocol or manual tracing, and (3) a novice rater who was unfamiliar with DTI, the protocol, and manual tracing. Then, the fornix FTL was computed using the FACT algorithm length distributions across the fornix following the main direction of its principal eigenvector in each individual using Euler's method (Yeh et al., 2013). Each completed the tracings three times within 1-month interval to determine intra- and inter-rater reliability of the manual tracings. The FEFA maps were calculated based on a combination of direction and anisotropic diffusion, represented in red, green, and blue. Figure 2 shows the manually traced tract (red line) drawn on the 2D FEFA map on the axial plane of the fornix (green color diffusion direction).

### Statistical Analysis

Statistical analyses were performed using IBM-SPSS version 26 (IBM Corp. Released 2019 from the manual tracing IBM SPSS Statistics for Windows, Version 26.0; IBM Corp., Armonk, NY). Independent *t*-tests were used to compare age and education level between the HC and n-aMCI groups. ANOVAs were used to compare groups on MoCA, attention, executive functioning, memory, and the DTI parameters; FTL from the manual tracing (FiberTrak) and (FA_whole_, ADC_whole_, FA_BC_, FA_ST_, ADC_BC_, and ADC_ST_) extracted from the JHU-atlas masks, all controlling for age and education level. Pearson correlation coefficients were calculated to determine the strength of the linear association among DTI parameters and cognitive composites (Supplementary Table 4). Separate hierarchical linear regressions for each DTI parameter were performed to predict the cognitive composites, controlling for age and education. To achieve this, the HC group was coded as 1 and the n-aMCI group as 2, and age and education were entered first. Because age and education correlated with FTL (Supplementary Table 4), the interactions of FTL with age and education were included as a second step to account for these relationships. Next entered was a DTI parameter (e.g., FA of the entire fornix) and then the group × DTI parameter interactions. The group × DTI interaction term allowed us to determine if the relationship between DTI parameters and cognition differed between groups over and above any effects of age or education. The alpha level was set at 0.05 throughout.

## Results

### Demographic Data

Table 1 shows the descriptive statistics of the demographic data. The HC group was significantly younger (64.36 ± 3.93 years) than the n-aMCI group (71.24 ± 8.15 years), *p* = 0.005, and significantly more educated (HC = 15.79 ± 2.61, n-aMCI = 12.18 ± 5.71), *p* = 0.029. Furthermore, age and education were correlated with cognition and with FTL from the manual tracing; Supplementary Table 4). Therefore, age and education were controlled in all remaining group comparisons.

**Table 1:** Descriptive statistics of participants' demographics.

**Variables**	**Mean** **±** **SD**		**Group comparison**	
	**HC**	**n-aMCI**	***F***	***p***
Subjects (*n)*	14	17	–	–
Age (years)	64.36 ± 3.93	71.24 ± 8.15	4.868	**0.005**
Education level (years)	15.79 ± 2.61	12.18 ± 5.71	1.713	**0.029**
Gender (M:F)	0:14	3:14	4.278	0.098

*Age and education level were compared using an independent t-test and a chi-square test, respectively. HCs, healthy older adult controls, n-aMCI, non-amnestic mild cognitive impairment (MCI) group. Significant values are bolded*.

### Qualitative Analysis

**Figure 3:**
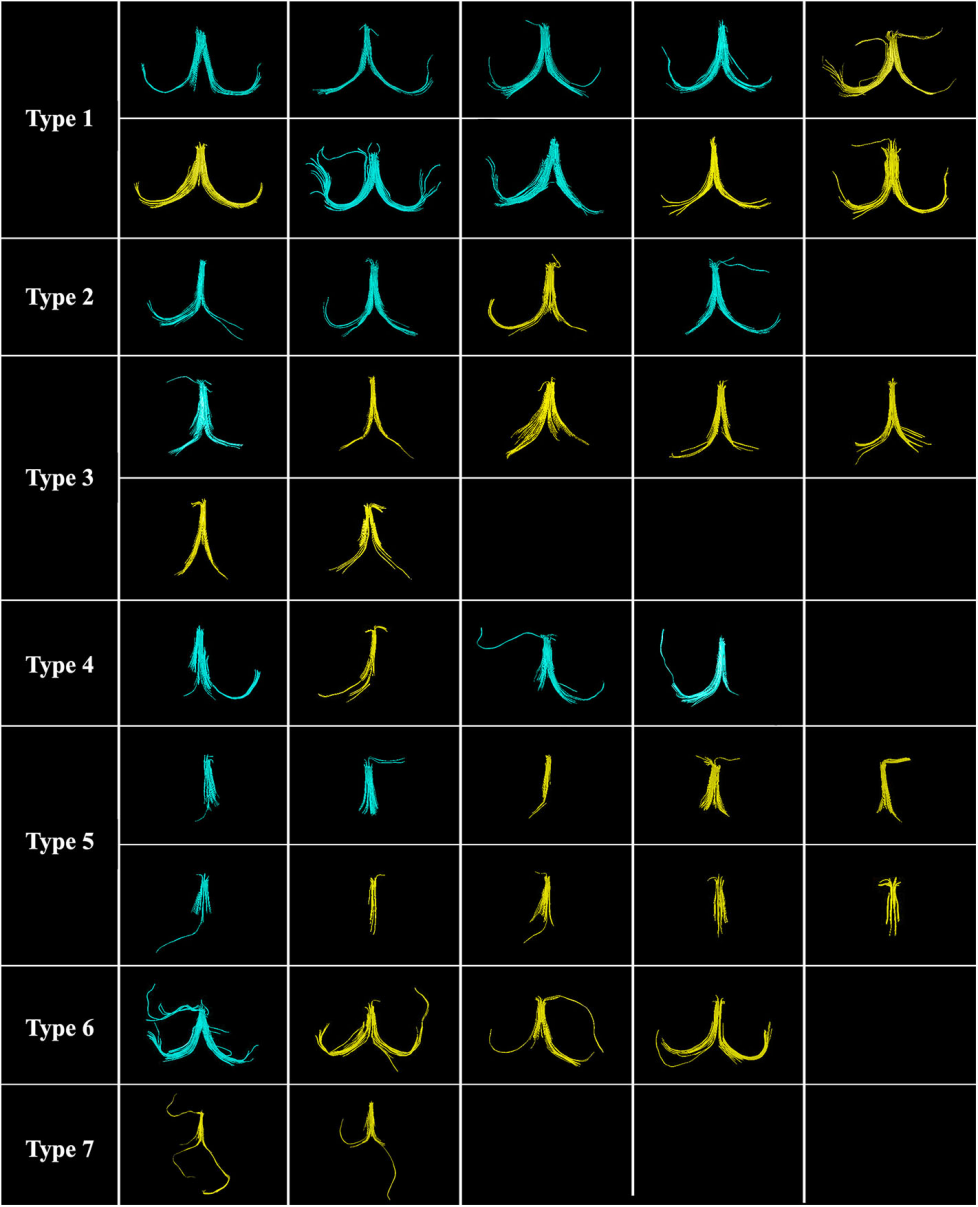
Seven classes of the fornix projection are classified by using a single region of interest (ROI) (*n* = 41). Ten cases (24.4%) of Type 1, the complete typical projection; four cases (9.8%) of Type 2, complete typical projection with short fimbria (one side, Lt/Rt); seven cases (17.1%) of Type 3, complete typical projection with short fimbriae (both sides, Lt. and Rt.); four cases (9.7%) of Type 4, atypical projection with missing crus and/or fimbria (one side, Lt/Rt); 10 cases (24.4%) of Type 5, atypical projection with missing both crura and fimbriae (both sides, Lt. and Rt.); four cases (9.8%) of Type 6, complete typical projection with addition atypical projection; and two cases (4.9%) of Type 7, the atypical projection of fimbriae (both sides, Lt. and Rt.). Six cases (14.6%) of Type 6 and Type 7 were excluded due to the atypical projection of the fornix tract. Note: Healthy control (HC) group is shown in blue, and non-amnestic mild cognitive impairment (MCI) group (n-aMCI) group is shown in yellow.

Although the anatomy of the fornix is well-established, substantial intersubject anatomical variability is observed. The 3D reconstruction of the manually labeled fornix tracts was classified into seven classes of projection based on a skilled neuroanatomist's knowledge of the typical complete projection of the fornix consisting of fimbriae, crura, body, and column. The seven classes identified included three types of typical projection, two types of typical projection with missing features, and two types of atypical projection (Figure 3). Six cases (14.6% of all cases) of Type 6 and Type 7 were excluded due to the atypical projection of the fornix tract.

### Intra- and Inter-rater Reliability

Intra-rater consistency of the manual tracing in fornix FTL ranged from acceptable to excellent, with Cronbach's alpha of 0.733 to 0.972, as shown in Table 2. Inter-rater consistency was excellent, with Cronbach's alpha ranging from 0.950 to 0.990. The inter-rater reliability of FTL in the HC group showed the highest reproducibility among both groups. In addition, the intra/inter-rater consistency averaged across all iterations and raters was excellent, with the highest Cronbach's alpha of 0.993. It was these latter averages that were brought forward for analysis.

**Table 2:** The internal consistency (Cronbach's alpha) of the manual tracing of the fornix, both intra-observer and among three levels of inter-observer including professional, intermediate, and novice levels.

**DTI parameters**	**Intra-rater consistency (α)**		**Inter-rater consistency (α)**		**Average of intra/inter-rater consistency (α)**	
	**HC (*n* = 14)**	**n-aMCI (*n* = 17)**	**HC (*n* = 14)**	**n-aMCI (*n* = 17)**	**HC (*n* = 14)**	**n-aMCI (*n* = 17)**
FTL (mm)	0.972	0.733	0.990	0.950	0.993	0.966

*HCs, healthy older adult controls; n-aMCI, non-amnestic mild cognitive impairment (MCI) group; FA, fractional anisotropy; ADC, apparent diffusion coefficient; FTL, fiber tract length*.

### Group Differences in Diffusion Tensor Imaging Parameters and Cognition

Descriptive statistics for cognitive screening scores, DTI parameters, and the z-scores of attention, executive function, and memory domains for each group are shown in Table 3. The HC group outperformed the n-aMCI group in the executive function and memory domains, but not in the attention domain, after controlling for age and education. No significant difference between groups was found in the age- and education-adjusted DTI parameters.

**Table 3:** Descriptive statistics of participants' MoCA score, cognitive composites, and diffusion tensor imaging (DTI) parameters.

**Variables**	**Mean** **±** **SD**		**Group comparison**	
	**HC**	**n-aMCI**	***F***	***p***
MoCA	26.07 ± 3.10	19.88 ± 4.06	7.804	**0.009**
Attention (z)	0.05 ± 0.46	−0.10 ± 0.44	0.000	0.988
Executive Function (z)	0.46 ± 0.39	−0.46 ± 0.44	19.703	**<0.001**
Memory (z)	0.24 ± 0.37	−0.19 ± 0.46	5.680	**0.024**
**JHU-atlas**				
FA_whole_	0.43 ± 0.03	0.42 ± 0.03	0.167	0.686
ADC_whole_	1.11 ± 0.09	1.15 ± 0.12	0.683	0.416
FA_BC_	0.33 ± 0.07	0.29 ± 0.08	2.366	0.136
ADC_BC_	1.71 ± 0.24	1.84 ± 0.30	1.722	0.200
**Manual tracing: FiberTrak**				
FTL (mm)	44.25 ± 9.66	37.59 ± 10.89	0.326	0.573

*The group comparisons were made using univariate ANOVA, controlling for age and education. HCs, healthy older adult controls; n-aMCI, non-amnestic mild cognitive impairment (MCI) group; MoCA, Montreal Cognitive Assessment; FA, fractional anisotropy; ADC, apparent diffusion coefficient; FTL, fornix fiber tract length; FA_BC_, FA value of the body and column of the fornix; FA_whole_, FA value of the whole fornix tract; ADC_BC_, ADC value of the body and column of the fornix; ADC_whole_, the ADC value of the whole fornix tract. Significant values are bolded*.

### Group Differences in the Relationship Between Diffusion Tensor Imaging Parameters and Cognition

Hierarchical linear regressions revealed no significant relationship of the whole-fornix FA or ADC with cognition. The same was true for the ADC of the BC of the fornix. However, significant relationships with cognition were identified in FA_BC_ and FTL, and these relationships furthermore differed between groups after accounting for the influence of age and education. FA_BC_ was positively associated with both executive function (*p* = 0.003) and memory (*p* = 0.035) overall, but more strongly in the HC than in the n-aMCI group (*p* < 0.001 and *p* = 0.028, respectively) (Table 4). While the association of FTL with executive function and memory was not significant overall, these relationships also differed between groups (*p* < 0.001 and *p* = 0.011, respectively) (Table 5). The association of FTL with memory was stronger in the HC group than that in the n-aMCI group, but contrary to the case of FA_BC_, the association of FTL with executive functioning was stronger in the n-aMCI group than that in the HC group. Importantly, these differences in the relationship between FA_BC_ and FTL with cognition were independent of any influence of age or education.

**Table 4:** Hierarchical linear regression analysis of fractional anisotropy (FA) of the body and column of the fornix and cognition among HC and non-amnestic mild cognitive impairment (MCI) group (n-aMCI) groups, with attention, executive function, and memory domains as dependent variables, adjusted for age and education.

**Model**	**Unstandardized coefficient**		**Standardized coefficient**	***t***	***p***	***F***	***R***	***R^**2**^***	**Δ*R*^**2**^**
	**B**	**SE**	**Beta**						
**1. Attention domain**									
Age	−0.005	0.013	−0.086	−0.413	0.683				
Edu	0.031	0.019	0.343	1.685	0.104				
FA_BC_	0.615	1.214	0.107	0.507	0.617				
FA_BC_*Group	0.057	0.598	0.023	0.095	0.925	1.244	0.401	0.161	<0.001
**2. Executive function domain**									
Age	−0.013	0.010	−0.159	−1.299	0.205				
Edu	0.042	0.015	0.330	2.770	**0.010**				
FA_BC_	3.199	0.990	0.401	3.230	**0.003**				
FA_BC_*Group	−2.097	0.488	−0.596	−4.298	**0.000**	16.169	0.845	0.713	0.204
**3. Memory domain**									
Age	0.013	0.012	0.210	1.136	0.266				
Edu	0.029	0.017	0.299	1.663	0.108				
FA_BC_	2.513	1.131	0.416	2.221	**0.035**				
FA_BC_*Group	−1.295	0.558	−0.487	−2.323	**0.028**	3.419	0.587	0.345	0.136

Edu, education level; FA_BC_, the FA value in the body and column of the fornix; FA_BC_*Group, group difference in FA_BC_. Significant values are bolded.

**Table 5:** Hierarchical linear regression analysis of fornix fiber length and cognition among HC and non-amnestic mild cognitive impairment (MCI) group (n-aMCI) groups, with attention, executive function, and memory domains as dependent variables, adjusted for age and education.

**Model**	**Unstandardized coefficient**		**Standardized coefficient**	***t***	***p***	***F***	***R***	***R^**2**^***	**Δ*R*^**2**^**
	**B**	**SE**	**Beta**						
**1. Attention domain**									
Age	−0.007	0.048	−0.108	−0.135	0.894				
Edu	−0.136	0.072	−1.495	−1.892	0.071				
Age * FTL	0.000	0.001	−0.509	−0.335	0.741				
Edu * FTL	0.005	0.002	3.052	2.443	**0.022**				
FTL	−0.53	0.100	−1.275	−0.530	0.601				
FTL * Group	0.002	0.004	0.116	0.571	0.573	2.323	0.606	0.367	0.209
**2. Executive function domain**									
Age	−0.022	0.042	−2.55	−0.515	0.612				
Edu	0.065	0.063	0.508	1.035	0.311				
Age * FTL	0.001	0.001	0.703	0.744	0.464				
Edu * FTL	−0.001	0.002	−0.353	−0.455	0.653				
FTL	0.005	0.086	0.086	0.057	0.955				
FTL * Group	−0.015	0.003	−0.569	−4.491	**<0.001**	12.374	0.869	0.756	0.695
**3. Memory domain**									
Age	0.012	0.049	0.194	0.252	0.803				
Edu	0.082	0.073	0.855	1.123	0.273				
Age * FTL	0.001	0.001	0.704	0.480	0.636				
Edu * FTL	−0.02	0.002	−0.999	−0.830	0.415				
FTL	0.020	0.101	0.465	0.200	0.843				
FTL * Group	−0.011	0.004	−0.540	−2.750	**0.011**	2.799	0.642	0.412	0.265

Edu, education level; FTL, fornix fiber tract length; Age * FTL, age by FTL interaction; Edu * FTL, education by FTL interaction; FTL * Group, group difference in FTL. Significant values are bolded.

## Discussion

### Demographics

All participants with MCI in our study were classified as n-aMCI with deficits in executive functioning. This is consistent with the high prevalence of cerebrovascular and cardiovascular conditions in the Asia-Pacific region compared to other regions (Jhoo et al., 2008; Chan et al., 2013), although it should be noted that n-aMCI can progress to AD, and individuals with AD often have mixed neuropathology that includes neurovascular events such as WM hyperintensities (Alber et al., 2019). Earlier studies have reported a higher prevalence of VaD than AD with an overall ratio of 2:1 in the Asia-Pacific population due to lifestyle and food preference (Narasimhalu et al., 2008). The HC group was significantly younger and had higher education levels than the n-aMCI group; therefore, all other group comparisons in this study accounted for these differences. Our findings in this regard are consistent with several studies that have demonstrated a link between educational attainment and cognitive functioning (Ardila et al., 2000; Le Carret et al., 2003; Narasimhalu et al., 2008; Falch and Sandgren Massih, 2011; Guerra-Carrillo et al., 2017).

### Fornix Projections and Integrity

DTI tractography was performed using the novel deterministic FACT algorithm. In the FACT algorithm, the fornix fiber tract is reconstructed voxel by voxel following the main direction of its principal eigenvector. Hence, this algorithm provided a reliable estimate of fornix FTL (Mori and Van Zijl, 2002; Hagler et al., 2009). Based on neuroanatomy knowledge, we found 35 of the 41 participants (85.37%) to have successfully represented the projection of fornix entirely and only six (9.76%) to have unsuccessful tracking results (presenting with the unlikely scenario of fiber projecting within subcortical WM) (Bürgel et al., 2009). For this reason, it was necessary to exclude these six aberrant fiber cases. Fornix WM integrity begins to decrease after its maturation peak during late adolescence because it is one of the earliest WM tracts to mature in the human brain (Douet and Chang, 2015). With advancing age, total WM fiber length in older adults has been reported to be decreased by 27 to 45% compared to younger adults (Tang et al., 1997; Marner et al., 2003). In our study, 14 cases had shortened or missing crura and/or fimbriae of the fornix including six cases (42.85%) of HC and eight cases (57.15%) of the n-aMCI group (as you can see in Figure 3; Type 4 and Type 5). Aging, together with the WM lesions (WMLs), in particular, specific frontal or medial temporal lobe (MTL) areas, could lead to a higher prevalence of atypical or incomplete fornix projection in n-aMCI than in the control group.

### The Relationship Between Fornix Integrity and Executive Function

Executive function (EF) encompasses higher-order cognitive processes that generally refer to the coordinated operation of organization, regulation, planning, working memory, problem-solving, cognitive flexibility, and cognitive fluency (Denckla, 1994; Alvarez and Emory, 2006; Chan et al., 2008). It has long been known that the PFC is a pivotal area for sustaining executive functioning (Welsh et al., 1991; Moriguchi and Hiraki, 2013; Yuan and Raz, 2014). As the fornix connects the limbic system with both prefrontal and subcortical regions, it is a critical component of the Papez circuit and serves a major efferent pathway from the hippocampus to the medial PFC. Indeed, executive dysfunction was found to be related to cerebral hypoperfusion in regions connected to the fornix, specifically the middle frontal cortex and posterior cingulate gyrus in people with n-aMCI and executive dysfunction (Chao et al., 2009). Moreover, infarction of the fornix also leads to amnesia with executive dysfunction (Rizek et al., 2013; Salvalaggio et al., 2018).

We found a significant relationship between executive function and integrity in the BC (FA_BC_) of the fornix as well as with fornix FTL, independent of the influence of age and education. Our results suggest that FA_BC_ does not support executive function as efficiently in n-aMCI compared to HC, as FA is less strongly associated with EF in the former group (Table 4). Interestingly, FTL was more strongly associated with EF in n-aMCI than the control group, and FTL was significantly positively related to EF over all participants (*r* = 0.60, *p* < 0.01; Supplementary Table 4). In n-aMCI, we found that most cases (57% of all n-aMCI cases) had missing crura and fimbriae, which are extended from the hippocampus. On the other hand, the BC of the fornix was intact in all cases. The column of the fornix projects to septal nuclei and the PFC *via* the precommissural fornix. As mentioned above, the PFC plays a critical role in executive functioning. Those with longer fornix FTL had better executive functioning, and this was particularly the case in the n-aMCI group. Given that the precommissural fornix projects to the PFC *via* the septal nuclei (Yeo et al., 2013; Cho et al., 2015; Coad et al., 2020), this finding suggests that, particularly among those with n-aMCI, executive functioning was sustained among those with fornix FTL long enough to make these connections.

### The Relationship Between Fornix Integrity and Memory

Episodic memory involves the ability to learn, store, and retrieve information (Dickerson and Eichenbaum, 2010). In our study, FA_BC_ and FTL both showed a significant positive relationship with memory performance, over and above the influences of age and education. Moreover, this relationship was stronger in the HC than in the n-aMCI group. These results replicate previous findings showing that fornix integrity is supportive of memory performance in healthy young and older adults (e.g., Rudebeck et al., 2009; Metzler-Baddeley et al., 2011) but also highlight the breakdown of the contribution of the fornix to episodic memory in individuals with n-aMCI. As is well known, the hippocampus–fornix–mammillary body system plays a role in episodic memory (Gaffan, 1992). Similar to hippocampus lesions, neurodegeneration of the fornix microstructure leads to the inability to create and/or store new memories (Thomas et al., 2011). This joint biological mechanism can potentially include Wallerian-like degeneration (WD) of the fornix axons, which is secondary to early injury of the neuronal degeneration in the hippocampus (Fletcher et al., 2013; Chen et al., 2017). The earliest study to our knowledge identifying WD in the fornix was after transection of the fimbria–fornix during temporal lobe epilepsy surgery for intractable epilepsy (Liu et al., 2013). More recently, Wang et al. (2020) reported that poorer fornix WM integrity was significantly correlated with reduced functional connectivity of the hippocampus due to the WD of the fornix axons in patients with MCI and AD (Wang et al., 2020). WD can be secondary to some cerebrovascular diseases (Uchino et al., 2004; Thomalla et al., 2005; Xie et al., 2012; Zhang et al., 2018), especially in the first week after ischemic stroke. It has been reported that the FA values of the affected tract begin to decrease 3 days after onset of the stroke (Thomalla et al., 2005; Xie et al., 2012; Zhang et al., 2018). Moreover, the fornix microstructure has been shown to predict episodic memory performance in several MRI studies (Vann et al., 2009; Sexton et al., 2010; Metzler-Baddeley et al., 2011; Zhuang et al., 2012).

Infarction of the fornix can lead to VaD or SVD, which also leads to a decline in memory performance (Cummings, 1994; Kalaria and Erkinjuntti, 2006). Likewise, the MTL is commonly affected by traumatic brain injury (TBI), which typically results in a variety of cognitive deficits. The pathophysiology of TBI is characterized by impaired regulation of cerebral blood flow (Werner and Engelhard, 2007; Prins et al., 2013), tissue damage involving the damage of limbic WM, and other factors such as edema, excitotoxicity, and hemorrhage (Gale et al., 1993). WM disruption in the fornix has been found to be associated with memory performance in both TBI patients and control groups (Kinnunen et al., 2011) and the reduction of FA of the fornix is correlated with poorer memory performance (Tomaiuolo et al., 2004), working memory (Palacios et al., 2011), and learning (Kinnunen et al., 2011) in patients with TBI.

In addition, anterograde amnesia, the inability to create new memories, is one of the earliest symptoms in patients with fornix infarction or after TBI that damages limbic-related structures including the fornix (Baweja et al., 2015; Gupta et al., 2015; Turine et al., 2016; Kauppila et al., 2018; Takano et al., 2018; Wang et al., 2018; Zhu et al., 2018). These results suggest that one contributor to episodic memory deficits in n-aMCI is the subtle degradation of fornix integrity.

### The Relationship Between Fornix Integrity and Attention

Attention refers to the ability to selectively attend or concentrate on specific relevant information while ignoring irrelevant information (McGuinness et al., 2010). The dorsolateral PFC and anterior cingulate gyrus are two areas involved in attention (Perry and Hodges, 1999). Because the fornix is one of the WM tracts carrying signals from the MTL to the PFC, damage to the fornix could lead to a decline in attention ability. Although our participants with n-aMCI had intact attention and there was no significant relationship between the DTI parameters and attention in the present study, several studies have found that patients with VaD have attentional deficits and more so than patients with AD (Mendez and Ashla-Mendez, 1991; Barr et al., 1992; Almkvist et al., 1993). Therefore, we might expect a significant decline in attention ability and its relationship with fornix integrity in the later stages of VaD in our sample. It is also the case that our measures of attention did not assess higher-order attention skills such as divided attention. Perhaps we would have seen group differences had we administered more complex attention tasks.

### Fornix and Its Association With Vascular Dementia

Changes in fornix diffusivity are common among patients with VaD (Douaud et al., 2011; Mayo et al., 2017; Salvadores et al., 2017), especially the reduction of FA and an increase of ADC, which reflect its integrity. The higher the frequency of ischemic heart disease, TIA, or stroke in a sample, the more WMLs are found in n-aMCI compared to aMCI patients (Mariani et al., 2007). Likewise, those with n-aMCI typically have increased vascular burden and are more likely to have cardiovascular risk factors as well as basal forebrain atrophy than those with aMCI (He et al., 2009; Jak et al., 2009). These vascular burdens, such as small ischemic and vascular lesions that involve subcortical areas (where the fornix is situated) are commonly associated with cognitive decline (Cummings, 1994). As is well known, n-aMCI is more likely to develop into non-AD dementia, notably VaD (Petersen et al., 2001).

### Limitations and Future Directions

One limitation of this pilot study is the small sample size, which may have limited the power of investigation. We initially recruited 80 participants but were very conservative in excluding conditions that might affect cognition, other than preclinical neurodegeneration. Thus, although our sample is small, we are highly confident in the clinical diagnosis of our sample. Another limitation of our data is that age and education are strongly associated with cognition; age and education differed between the patient and control groups. However, we have shown that the significance relationship between cognition and DTI parameters in our study is independent of any influence of age or education. Another limitation is the deterministic tractography, which can only detect local diffusion that passes through the chosen ROI and is unable to distinguish between afferent and efferent fibers within the WM tract; hence, it cannot be assumed that the detected projection reflects the true anatomical structure (Mori et al., 1999; Bürgel et al., 2009).

Because the fornix is small and located between the lateral ventricles beneath the corpus callosum along with septum pellucidum, it is susceptible to partial volume effects by the surrounding CSF, which can potentially affect the measurement of the thinner parts of it (i.e., crura and fimbriae). By contrast, the BC of the fornix is the most prominent structure, making it least susceptible to partial volume effects and thus a good candidate for representing the WM integrity of the entire fornix.

## Conclusion

The FA of the BC of the fornix and fornix FTL were positively associated with executive function and memory among both groups. These relationships were stronger in the healthy older adults than those in the n-aMCI, with the exception of the FTL–executive functioning association. This pilot study provides the first evidence for a decline in the contributions of fornix integrity to memory and executive functioning in n-aMCI and suggests that maintenance of fornix FTL is critical for sustaining executive functioning in people with presumptive preclinical VaD.

## Data Availability Statement

The raw data supporting the conclusions of this article will be made available by the authors, without undue reservation.

## Ethics Statement

This study was reviewed and approved by Research Ethics Committee of the Faculty of Medicine, Chiang Mai University. The patients/participants provided their written informed consent to participate in this study.

## Author Contributions

JC and SK contributed to the provision of software and computational resources. JC, NW, NA, PV, PM, PS, and SK contributed to the study design. JC, KU, NW, NA, PV, PM, and SK contributed to the review and editing of the manuscript. JC contributed to the supervision of imaging analysis. NW contributed to the supervision of investigation-clinical aspects of the work, field mentorship, and ethics. NA contributed to the supervision of statistical analysis and clinical aspects of the work. PM contributed to the supervision and project administration. SK contributed to the supervision of DTI and MRI. NW, PV, and PS contributed to the data collection. PM and PS obtained funding for the study. PS contributed to the conceptualization and writing the original draft. All authors contributed to the article and approved the submitted version.

## Conflict of Interest

The authors declare that the research was conducted in the absence of any commercial or financial relationships that could be construed as a potential conflict of interest.
