# Supplementary material for: Fornix Integrity Is Differently Associated With Cognition in Healthy Aging and Non-amnestic Mild Cognitive Impairment: A Pilot Diffusion Tensor Imaging Study in Thai Older Adults
Source: Front Aging Neurosci. 2020 Dec 2;12:594002. doi: 10.3389/fnagi.2020.594002 (PMC7745667; doi:10.3389/fnagi.2020.594002)
Supplement: Supplementary file 1 [file Data_Sheet_1.docx]

Supplementary Material

1. **Supplementary Table 1.** Hierarchical linear regression analysis of FA of the whole tract of fornix and cognition among CN and n-aMCI groups with attention, executive function, and memory domains as dependent variables, with age and education level as the covariates.
2. **Supplementary Table 2.** Hierarchical linear regression analysis of ADC of the whole tract of fornix and cognition among CN and n-aMCI groups with attention, executive function, and memory domains as dependent variables, with age and education level as the covariates.
3. **Supplementary Table 3.** Hierarchical linear regression analysis of ADC of the body and column of the fornix and cognition among CN and n-aMCI groups with attention, executive function, and memory domains as dependent variables, with age and education level as the covariates.
4. **Supplementary Table 4.** Correlation (r) among age, education, cognitive domains, and DTI parameters.

**Supplementary Table 1.** Hierarchical linear regression analysis of FA of the whole tract of fornix and cognition among CN and n-aMCI groups with attention, executive function, and memory domains as dependent variables, with age and education level as the covariates. Note: Edu = education level; FA_whole_ = the FA value of the whole tract of fornix; FA_whole_*Group = group difference in FA_whole_. Significant values are bolded.

| **Model** | **Unstandardized coefficient** | | **Standardized coefficient** | **t** | **p** | **F** | **R** | **R2** | **∆R2** |
| --- | --- | --- | --- | --- | --- | --- | --- | --- | --- |
|  | **B** | **SE** | **Beta** |  |  |  |  |  |  |
| **1. Attention domain** | | | | | | | | | |
| Age | -0.005 | 0.013 | -0.081 | -0.38 | 0.707 |  |  |  |  |
| Edu | 0.031 | 0.019 | 0.342 | 1.683 | 0.104 |  |  |  |  |
| FA_whole_ | -0.136 | 2.517 | -0.01 | -0.054 | 0.957 |  |  |  |  |
| FA_whole_*Group | -0.016 | 0.444 | -0.008 | -0.037 | 0.971 | 1.116 | 0.383 | 0.147 | <0.001 |
| **2. Executive Function domain** | | | | | | | | | |
| Age | -0.012 | 0.01 | -0.145 | -1.176 | 0.250 |  |  |  |  |
| Edu | 0.045 | 0.015 | 0.352 | 2.993 | **0.006** |  |  |  |  |
| FA_whole_ | 2.693 | 2.032 | 0.143 | 1.325 | 0.197 |  |  |  |  |
| FA_whole_*Group | -1.591 | 0.359 | -0.548 | -4.437 | **0.000** | 16.24 | 0.845 | 0.714 | 0.216 |
| **3. Memory domain** | | | | | | | | | |
| Age | 0.015 | 0.012 | 0.231 | 1.229 | 0.230 |  |  |  |  |
| Edu | 0.031 | 0.017 | 0.323 | 1.799 | 0.084 |  |  |  |  |
| FA_whole_ | 2.701 | 2.345 | 0.19 | 1.152 | 0.260 |  |  |  |  |
| FA_whole_*Group | -0.993 | 0.414 | -0.452 | -2.399 | **0.024** | 3.258 | 0.578 | 0.334 | 0.148 |

**Supplementary Table 2.** Hierarchical linear regression analysis of ADC of the whole tract of fornix and cognition among CN and n-aMCI groups with attention, executive function, and memory domains as dependent variables, with age and education level as the covariates. Note: Edu = education level; ADC_whole_ = the ADC value of the whole tract of fornix; ADC_whole_*Group = group difference in ADC_whole_. Significant values are bolded.

| **Model** | **Unstandardized coefficient** | | **Standardized coefficient** | **t** | **p** | **F** | **R** | **R2** | **∆R2** |
| --- | --- | --- | --- | --- | --- | --- | --- | --- | --- |
|  | **B** | **SE** | **Beta** |  |  |  |  |  |  |
| **1. Attention domain** | | | | | | | | | |
| Age | -0.005 | 0.013 | -0.089 | -0.42 | 0.678 |  |  |  |  |
| Edu | 0.032 | 0.018 | 0.345 | 1.717 | 0.098 |  |  |  |  |
| ADC_whole_ | 0.084 | 0.843 | 0.021 | 0.1 | 0.921 |  |  |  |  |
| ADC_whole_*Group | 0.009 | 0.165 | 0.013 | 0.055 | 0.956 | 1.121 | 0.384 | 0.147 | <0.001 |
| **2. Executive Function domain** | | | | | | | | | |
| Age | -0.013 | 0.011 | -0.15 | -1.203 | 0.240 |  |  |  |  |
| Edu | 0.049 | 0.015 | 0.382 | 3.227 | **0.003** |  |  |  |  |
| ADC_whole_ | 0.635 | 0.693 | 0.111 | 0.916 | 0.368 |  |  |  |  |
| ADC_whole_*Group | -0.56 | 0.136 | -0.566 | -4.129 | **0.000** | 15.447 | 0.839 | 0.704 | 0.194 |
| **3. Memory domain** | | | | | | | | | |
| Age | 0.014 | 0.012 | 0.212 | 1.12 | 0.273 |  |  |  |  |
| Edu | 0.034 | 0.017 | 0.356 | 1.98 | 0.058 |  |  |  |  |
| ADC_whole_ | 0.019 | 0.795 | 0.004 | 0.024 | 0.981 |  |  |  |  |
| ADC_whole_*Group | -0.325 | 0.156 | -0.434 | -2.09 | **0.047** | 3.034 | 0.564 | 0.318 | 0.114 |

**Supplementary Table 3.** Hierarchical linear regression analysis of ADC of the body and column of the fornix and cognition among CN and n-aMCI groups with attention, executive function, and memory domains as dependent variables, with age and education level as the covariates. Note: Edu = education level; ADC_BC_ = the ADC value of the body and column of the fornix; ADC_BC_*Group = group difference in ADC_BC_. Significant values are bolded.

| **Model** | **Unstandardized coefficient** | | **Standardized coefficient** | **t** | **p** | **F** | **R** | **R2** | **∆R2** |
| --- | --- | --- | --- | --- | --- | --- | --- | --- | --- |
|  | **B** | **SE** | **Beta** |  |  |  |  |  |  |
| **1. Attention domain** | | | | | | | | | |
| Age | -0.006 | 0.013 | -0.095 | -0.446 | 0.660 |  |  |  |  |
| Edu | 0.032 | 0.018 | 0.351 | 1.759 | 0.090 |  |  |  |  |
| ADC_BC_ | -0.144 | 0.382 | -0.09 | -0.378 | 0.709 |  |  |  |  |
| ADC_BC_*Group | 0.02 | 0.107 | 0.051 | 0.192 | 0.850 | 1.158 | 0.389 | 0.151 | <0.001 |
| **2. Executive Function domain** | | | | | | | | | |
| Age | -0.013 | 0.011 | -0.15 | -1.173 | 0.251 |  |  |  |  |
| Edu | 0.05 | 0.015 | 0.39 | 3.258 | **0.003** |  |  |  |  |
| ADC_BC_ | 0.591 | 0.32 | 0.266 | 1.851 | 0.076 |  |  |  |  |
| ADC_BC_*Group | -0.356 | 0.089 | -0.635 | -3.987 | **0.000** | 14.759 | 0.833 | 0.694 | 0.187 |
| **3. Memory domain** | | | | | | | | | |
| Age | 0.013 | 0.012 | 0.204 | 1.061 | 0.299 |  |  |  |  |
| Edu | 0.035 | 0.017 | 0.36 | 1.995 | 0.057 |  |  |  |  |
| ADC_BC_ | 0.175 | 0.364 | 0.104 | 0.48 | 0.635 |  |  |  |  |
| ADC_BC_*Group | -0.199 | 0.102 | -0.469 | -1.958 | 0.061 | 2.871 | 0.553 | 0.306 | 0.102 |

**Supplementary Table 4.** Correlation (r) among age, education, cognitive domains, and DTI parameters. Note: FA_w = the FA value of the whole tract of the fornix; ADC_w = the ADC value of the whole tract of the fornix; FA_BC = the FA value of the body and column of the fornix; FA_ST = the FA value of the stria terminalis; ADC_BC = the ADC value of the body and column of the fornix; ADC_ST = the ADC value of the stria terminalis; FTL = the fornix fibre tract length. Values significant at the level p<.01 are bolded. *p<.05, **p<.01, ***p<.001

|  | **Age** | **Edu** | **Atten** | **ExecFx** | **Mem** | **FA_w** | **ADC_w** | **FA_BC** | **FA_ST** | **ADC_BC** | **ADC_ST** | **FTL** |
| --- | --- | --- | --- | --- | --- | --- | --- | --- | --- | --- | --- | --- |
| **Age** | 1 |  |  |  |  |  |  |  |  |  |  |  |
| **Edu** | -.380* | 1 |  |  |  |  |  |  |  |  |  |  |
| **Atten** | -0.213 | .375* | 1 |  |  |  |  |  |  |  |  |  |
| **ExecFx** | **-.541**** | **.622**** | 0.318 | 1 |  |  |  |  |  |  |  |  |
| **Mem** | -0.118 | .415* | 0.221 | **.602**** | 1 |  |  |  |  |  |  |  |
| **FA_w** | -0.144 | 0.035 | 0.012 | 0.115 | 0.117 | 1 |  |  |  |  |  |  |
| **ADC_w** | 0.085 | 0.006 | 0.021 | -0.146 | -0.164 | **-.929**** | 1 |  |  |  |  |  |
| **FA_BC** | -0.047 | 0.065 | 0.144 | 0.167 | 0.211 | **.904**** | **-.931**** | 1 |  |  |  |  |
| **FA_ST** | -0.206 | -0.004 | -0.119 | 0.042 | 0.003 | **.913**** | **-.760**** | **.652**** | 1 |  |  |  |
| **ADC_BC** | 0.06 | -0.035 | -0.077 | -0.147 | -0.185 | **-.880**** | **.950**** | **-.972**** | **-.636**** | 1 |  |  |
| **ADC_ST** | 0.103 | 0.053 | 0.134 | -0.125 | -0.117 | **-.860**** | **.925**** | **-.755**** | **-.807**** | **.759**** | 1 |  |
| **FTL** | **-.734**** | .422* | 0.257 | **.600**** | 0.277 | 0.179 | -0.085 | 0.055 | 0.264 | -0.08 | -0.08 | 1 |
